# Complete Genome of Sinorhizobium meliloti AK76, a Symbiont of Wild Diploid Medicago lupulina from the Mugodgary Mountain Region

**DOI:** 10.1128/mra.01088-21

**Published:** 2022-02-28

**Authors:** Maria E. Vladimirova, Victoria S. Muntyan, Alexey M. Afonin, Alexey N. Muntyan, Olga A. Baturina, Elena A. Dzuybenko, Alla S. Saksaganskaya, Boris V. Simarov, Marina L. Roumiantseva, Marsel R. Kabilov

**Affiliations:** a Federal State Budget Scientific Institution All-Russian Research Institute for Agricultural Microbiology (FSBSI ARRIAM), Laboratory of Genetics and Selection of Microorganisms, Saint Petersburg, Russian Federation; b Institute of Chemical Biology and Fundamental Medicine, Siberian Branch of the Russian Academy of Sciences (ICBFM SB RAS), Novosibirsk, Russian Federation; c Vavilov All-Russian Institute of Plant Genetic Resources (VIR), Saint Petersburg, Russian Federation; SIPBS, University of Strathclyde

## Abstract

Sinorhizobium meliloti is a symbiotic bacterial species forming nitrogen-fixing nodules on roots of annual and perennial *Medicago* spp. We report the full genome sequence of S. meliloti strain AK76, an effective symbiont of the wild diploid plant Medicago lupulina grown in the Mugodgary Mountain region, Kazakhstan.

## ANNOUNCEMENT

Sinorhizobium meliloti AK76 was isolated from pink nodules of wild-growing annual diploid Medicago lupulina native to the Mugodgary Mountains at the northwest of Kazakhstan (48.58°N, 57.7697°E) in 2002 as a strain tolerant to 0.75 M NaCl (1–3). *Medicago lupulina* plants are well nodulated by two closely related rhizobial species, one of which is Sinorhizobium medicae (4). Here, we report the genome of S. meliloti native isolate AK76.

The AK76 strain was isolated from sterilized crushed well-developed pink nodules and passaged several times on tryptone-yeast (TY) medium (28°C) in order to get pure single colonies, which were further long-term stored at −80°C (1, 5). To rejuvenate AK76, its cells were restarted on solid TY plates, and then a single colony was grown in liquid TY (28°C, 180 rpm) up to an optical density at 600 nm of >0.75. Cells were harvested by centrifugation at 4,000 × *g*. Genomic DNA was isolated using phenol-chloroform extraction (6).

Genomic DNA was isolated with the GeneJET genomic DNA purification kit (Thermo Fisher Scientific) and fragmented to an average size of 600 bp in a microTUBE AFA fiber snap-cap tube (Covaris S2 instrument). The paired-end (PE) library was constructed using dual-index NEBNext multiplex oligonucleotides and a NEBNext Ultra II DNA library prep kit for Illumina (New England BioLabs). The DNA library was sequenced with a reagent kit v3 (600 cycle) on a MiSeq sequencer (Illumina) in the Genomics Core Facility (Institute of Chemical Biology and Fundamental Medicine, Siberian Branch of the Russian Academy of Sciences [ICBFM SB RAS]), and 4.4 million PE reads were generated.

Default parameters were used for all software unless otherwise specified. The reads were quality trimmed, and adapter sequences were removed using Trim Galore v0.4.4 (https://github.com/FelixKrueger/TrimGalore). Long reads for isolated AK76 DNA were obtained using a MinION (Oxford Nanopore) sequencer R9.4 (Federal State Budget Scientific Institution All-Russian Research Institute for Agricultural Microbiology [FSBSI ARRIAM]). A barcoded DNA library constructed with the 1D native barcoding genomic DNA protocol (with kits EXP-NBD103 and SQK-LSK108). The raw fast5 files were base called with Albacore v2.3.1 (the run yielded 307,000 reads with an *N*_50_ of 7 kb). The resulting reads were demultiplexed using Deepbinner v0.2.0 (7) and cleaned using Porechop v0.2.3 (https://github.com/rrwick/Porechop).

Illumina and Nanopore reads were assembled into 4 replicons—chromosome (3.58 Mb; GC content, 62.85%), pSymA (1.58 Mb; GC content, 60.29%), pSymB (1.72 Mb; GC content, 62.28%), and pAK76 (172.8 kb; GC content, 59.11%)—by Trycycler v0.4.1 (8). The complete genome size of AK76 is 7.05 Mb. Genome annotation of AK76 by NCBI Prokaryotic Genome Annotation Pipeline (PGAP) v5.0 (9) resulted in 6,650 coding DNA sequences (CDS), including sequences encoding 6,296 proteins, 68 RNAs (tRNA, transfer-messenger RNA [tmRNA], noncoding RNA [ncRNA], and rRNA of the 3 *rrn* operons), and 286 pseudogenes. A single genomic island (40.29 kb; GC content, 58.47%) containing 53 open reading frames (ORFs) was detected on the AK76 chromosome using Islander v0.1 (10). PHASTER web server (accessed 14 September 2021) analysis (11) indicated that 16 of these ORFs were homologous to the intact *Rhizobium* phage 16-3 (GenBank accession number NC_011103). GTDB-tk v1.7 (12) with reference data for Genome Taxonomy Database (GTDB) R202 were used for taxonomic classifications of the genome. Analysis confirmed that the AK76 strain is Sinorhizobium meliloti and the closest strain to AK76 is USDA1002 (GenBank assembly accession number GCA_009601385).

### Data availability.

The genome sequence of Sinorhizobium meliloti AK76 is deposited in GenBank under accession numbers CP066358, CP066359, CP066360, and CP066361. The raw reads are available in SRA under the accession numbers SRX13025480 and SRX13025481. This announcement describes the first version of the genome assembly.
